# α‐Synuclein in blood cells differentiates Parkinson’s disease from healthy controls

**DOI:** 10.1002/acn3.50944

**Published:** 2019-11-19

**Authors:** Suaad Abd Elhadi, Jessica Grigoletto, Maura Poli, Paolo Arosio, David Arkadir, Ronit Sharon

**Affiliations:** ^1^ Department of Biochemistry and Molecular Biology IMRIC The Hebrew University‐Hadassah Medical School Ein Kerem 9112001 Jerusalem Israel; ^2^ Laboratory of Molecular Biology Department of Molecular and Translational Medicine University of Brescia Brescia Italy; ^3^ Department of Neurology Hadassah‐Hebrew University Medical Center Jerusalem Israel

## Abstract

**Objective:**

To determine whether blood cells expressed α‐Syn can differentiate Parkinson’s disease (PD) from healthy controls (HC).

**Methods:**

The concentrations of α‐Syn were determined in samples of blood cell pellets using a quantitative Lipid‐ELISA assay. In addition, the levels of total protein, hemoglobin, iron and H‐ferritin were determined. The study includes samples from the Biofind cohort (*n* = 46 PD and 45 HC) and results were validated with an additional cohort (*n* = 35 PD and 28 HC).

**Results:**

A composite biomarker consisting of the concentrations of total α‐Syn, proteinase‐K resistant (PK^res^) α‐Syn and phospho‐Serine 129 α‐Syn (PSer 129), is designed based on the analysis of the discovery BioFIND cohort. This composite biomarker differentiates a PD subgroup, presenting motor symptoms without dementia from a HC group, with a convincing accuracy, represented by an AUC = 0.81 (95% CI, 0.71 to 0.92). Closely similar results were obtained for the validation cohort, that is, AUC = 0.81, (95% CI, 0.70 to 0.94).

**Interpretation:**

Our results demonstrate the potential usefulness of blood cells expressed α‐Syn as a biomarker for PD.

## Introduction

The complex etiology of Parkinson’s disease (PD) is only poorly understood. A growing evidence now suggests that neurodegeneration in PD is not restricted to the dopaminergic neurons localizing to the substantia nigra. Rather, that PD is a systemic disease, involving peripheral tissues and may include oxidative, metabolic, inflammatory, or biochemical processes.^1^

PD is a heterogeneous disease with a spectrum of motor and non‐motor features^2^ and may include subtypes. Yet, although recognized, a classification for subtypes is missing or incomplete.^3^, ^4^ Subtypes classification may potentially involve presentation of cognitive symptoms in addition to the classical disease motor symptoms.

The pathological hallmark of PD is the occurrence of Lewy pathology in the central nervous system (CNS). Lewy pathology also occurs in the peripheral nervous system,^5^ in neurons of the gastrointestinal tract^6^ and in the appendix.^7^ α‐Synuclein (α‐Syn) is a major constituent of Lewy pathology.^8^, ^9^ An ordered and predicted disease propagation path, which affects the peripheral nervous system and propagates to the CNS, fits with Braak’s hypothesis^6^, ^10^, ^11^, ^12^ and highlights a role for peripheral α‐Syn in the pathogenesis of PD.

The accessibility of the blood makes it a favorable sampling bio‐fluid that can assist the follow‐up and treatment of a patient during the course of the disease. α‐Syn in blood has been tested as a biomarker for PD.^13^, ^14^, ^15^, ^16^, ^17^, ^18^ However, it is important to emphasize the biology of α‐Syn in the blood and the potential relevance to the disease. A principal source for α‐Syn detected in blood is blood cells expressed α‐Syn, particularly of erythroid lineage.^19^, ^20^, ^21^ In addition, low levels of a neuronal‐expressed, prion‐like secreted α‐Syn, may be found in blood plasma.^15^, ^16^ Neuronal‐secreted α‐Syn represents a form that is associated with the pathogenic spread of the disease. However, the relevance of blood‐cells expressed α‐Syn to the pathogenesis of the disease is not fully clear yet. Blood cells‐expressed α‐Syn is mostly contributed by red blood cells.^19^ Platelets and blood mononuclear cells also express α‐Syn, however, at lower levels.^19^, ^22^

We determined the levels of total α‐Syn, proteinase K‐resistant (PK^res^) α‐Syn, phospho Serine 129 (PSer 129) α‐Syn and oxidized α‐Syn, in samples of blood cell pellets. In addition, we determined the levels of total protein, hemoglobin, iron and H‐ferritin. Based on the concentrations of total, PK^res^ and PSer129 α‐Syn we developed a logistic regression model for PD diagnosis. The results obtained in a BioFIND discovery cohort, comprising PD and healthy control (HC) donors, were validated in a second cohort. The results show that α‐Syn detected in blood cells differentiates PD patients presenting with motor symptoms without dementia and HC with a considerable degree of accuracy.

## Methods

### Samples and preparation

BioFIND^23^ samples comprising whole blood cell pellets arrived frozen on dry ice. Samples were thawed, divided in aliquots and kept frozen at −80°C until used. Aliquots were thawed one more time for analysis. Leftovers of a thawed aliquot were discarded. Samples of 5 mL blood in EDTA blood collection tubes were obtained from the neurology department at Hadassah hospital. Blood samples were processed within 1–2 h from collection. Blood was spun at 2000*g*, plasma was removed and whole blood cell‐pellets were kept in frozen aliquots. Blood cells were lysed by osmosis in 1:5 volumes of cold double distilled water (DDW). Lysed blood cells were spun at 17,000*g*, at 4°C for 30 min. The supernatant was used for measurements within one day from thawing an aliquot. Samples were shuffled and randomly assigned for analysis. Each microtitter plate contained an equal number of HC and PD samples. All measurements were performed blinded to group identity.

### Ethics statement and participant recruitment

Blood samples were collected with the informed consent of participant donors. The study was approved by the institutional Ethics Committee (0133‐16‐HMO and 0346‐17‐HMO). For additional ethic considerations see.^23^

Iron: determined as described previously.^24^ Briefly, acid extracted sample was added to a chromogen reagent (1 volume of 0.1% bathophenanthroline sulfate and 1% thioglycolic acid solution; 5 volumes of water; and 5 volumes of saturated sodium acetate). Absorbance determined at 535 nm in parallel to known amounts of iron as a standard curve.

Hemoglobin: measurements performed using the Triton X‐100/NaOH method 25, in which hemoglobin is converted to a colorimetric product that is determined at 574 nm. The concentration of hemoglobin was calculated according to a standard curve consisting of 0–2 μg/μL Hemin (Sigma, Rehovot, Israel).

H‐ferritin: determined by sandwich‐ELISA, with a monoclonal antibody rH02 as described previously.^26^ A standard curve consisting of a recombinant homo‐polymer H ferritin was used for reference amount. Of note, L‐ ferritin levels in blood cell pellets were below the limit of detection.

### α‐Syn detection by Lipid‐ELISA

A PolySorp, 96‐wells ELISA plate (Thermo Scientific, Getter, Israel) was coated with phosphatidylinositol (PI), phosphatidylserine (PS), phosphatidylethanolamine (PE) and GM‐1 ganglioside (Sigma, Rehovot, Israel) at 1:1:1:1 w/vol. Lipids were dissolved either in methanol or cyclohexene and applied in a final amount of 5 μg total lipids per well. Plates were incubated overnight at 4 ºC to complete evaporation of the solvent (immobilization of lipids). Blocking with 100 μl/well of 1% BSA (fatty acid‐free) in PBS (MgCl_2_ and CaCl_2_ free) for one hour at 37 ºC, followed by one wash with PBS. Protein levels were determined according to the Bradford method.^27^ Test samples were applied to the wells at the indicated protein amounts, in triplicates and incubated for 3 h at 37°C to allow capture of proteins by the immobilized lipids. The wells were washed with 3% H_2_O_2_ in DDW for 10 min and then with 1% formalin in PBS (without MgCl_2_ or CaCl_2_). A primary anti α‐Syn antibody was added to the wells, as indicated (below). Following incubation for one hour at 37°C, the wells were washed three times and either processed for detection (primary antibody conjugated to HRP) or incubated with a secondary antibody for one hour at 37°C diluted 1:8000 in 1% BSA in PBS and washed three times as above. Detection reaction as specified for each tested α‐Syn form. The amounts of α‐Syn were determined for each plate and each α‐Syn form according to the linear phase of a standard curve consisting of a recombinant α‐Syn protein, performed in parallel to the test samples.

#### Total α‐Syn

Samples of lysed blood cells were diluted to a final protein concentration of 0.8 µg/µl in 1% BSA (fatty acid free) in PBS (when immobilized lipids were dissolved in methanol, e.g., analyses 1 and 2) or in 0.1M sodium phosphate pH 8 (when immobilized lipids were dissolved in cyclohexene, e.g., analysis 3). Total α‐Syn was determined using anti α‐Syn ab MJF‐1 (Abcam, Tel‐Aviv, Israel) ‐ HRP conjugated. The detection step comprised addition of 100 μL of TMB one component microwell substrate (Southern Biotech, Birmingham, Alabama, USA) per well. The reaction was terminated with 100 μL/well of 1M H_2_SO_4_. Absorbance at 450 nm was determined using a plate‐reader (EL808 Ultra Microplate Reader, Bio‐Tek Instruments, VT, USA). Standard curve with 0–10 ng purified human wt α‐Syn. Assay sensitivity and specificity were described previously.^28^

#### PK^res^ α‐Syn

Twelve mg proteins of lysed blood cell were incubated with PK (0.6 μg/μL; #3115887001, Sigma, Rehovot, Israel) in homogenization buffer [20 mmol/L HEPES pH 7.3; 1 mmol/L MgCl_2_, 0.32 mol/L sucrose; 43 mmol/L β‐ME] as described previously.^17^ Following 25 min of incubation at 37°C, samples were transferred to 90°C for 10 min, to inactivate the protease and spun at 17,000*g* for 30 min. PK^res^ α‐Syn levels were determined using anti α‐Syn ab MJF‐1 (Abcam, Tel‐Aviv, Israel) ‐ HRP conjugated and detection reaction with TMB as above. Assay sensitivity and specificity were described previously.^28^

PSer129 α‐Syn: detection following hemoglobin clearance with HemoVoid kit (Biotech Support Group LLC, NJ, US). Samples of 4 mg lysed blood cells in homogenization buffer containing 1 mmol/L PMSF and 1 mmol/L NaF were applied into a tube containing 50 mg HemoVoid matrix. The eluted fraction was diluted 1:2 in 0.1 mol/L sodium‐phosphate pH 8 and applied at 0.16 mg (the equivalent amount of protein before HemoVoid clearance) per well on white PolySorp ELISA plates (Thermo Scientific, Getter, Israel) precoated with lipids dissolved in cyclohexene. Samples reacted with anti PSer129 α‐Syn monoclonal antibody (pSyn#64, WAKO, Osaka, Japan). A standard curve consisting of recombinant protein, phosphorylated at Ser129 (0–20 pg; Proteos, Kalamazoo, US), was applied in parallel to the test samples and used as a reference. Luminata Crescendo ELISA HRP substrate (Mercury, Rosh Ha’in, Israel) was used for the enzymatic reaction. Luminescence was determined by a luminometer (Infinite M200 Pro. NEOTEL Scientific Instrumentation Ltd.) immediately after adding the substrate. Assay sensitivity and specificity are described in Appendix S1 and Figure S1A,B.

#### Oxidized α‐Syn

Test samples treated with HemoVoid (see above) and applied on microtiter plate, pre‐coated with lipids dissolved in cyclohexene. Detection using anti oxidized α‐Syn ab, syn 303 (Biolegend, ENCO, Petach Tikvah, Israel). Detection performed with TMB reaction (as above). A standard curve consisting of a recombinant α‐Syn protein that was oxidized by ferrous sulfate (0.5 mmol/L) and H_2_O_2_ (1 mmol/L) 29 was applied in each plate as a reference. Assay sensitivity and specificity are described in Appendix S1 and Fig S1C,D.

#### Analyses of the discovery BioFIND cohort

Analysis 1: Total α‐Syn determined directly, at three different protein amounts: 8, 16, and 32 μg/well, in triplicates. PK^res^ α‐Syn, determined in samples pretreated with 0.6 μg/μl PK. Samples applied to the wells at 40, 80, 120 µg proteins (equivalent protein amount before PK treatment) in 1% BSA in PBS, in triplicates. A total of 4 different samples per a 96 wells plate. The immobilized lipids were dissolved in methanol.

Analysis 2: Total α‐Syn determined directly in test samples comprising 16 μg/well of total proteins, from 12 different test samples, applied in triplicates. PK^res^ α‐Syn applied at 80 µg proteins (equivalent protein amount before the PK treatment) in 1% BSA in PBS, in triplicates. A total of 12 different test samples were applied per 96‐well plate. The immobilized lipids were dissolved in methanol.

Analysis 3: Total α‐Syn and PK^res^ α‐Syn determined as in analysis 2. Immobilized lipids dissolved in cyclohexane.

Analysis 4: Samples of lysed blood cells (4 mg protein) were pre‐treated with 50 mg HemoVoid resin, according to manufacturer instructions (Biotech support group). For PSer129 α‐Syn or oxidized α‐Syn detection, samples of 160 μg proteins (an equivalent amount before the HemoVoid treatment) in 0.1M sodium phosphate pH 8, were applied in triplicates. A total of 24 test samples were applied per 96‐well microtiter plate. The immobilized lipids were dissolved in cyclohexane.

#### Analysis of the validation cohort

Total and PK^res^ α‐Syn levels were determined as described in analysis 2. PSer129 α‐Syn levels were determined as in analysis 4.

### Statistical analyses

The BioFIND groups (discovery cohort) were compared by Kruskal–Wallis test as implemented by the PMCMRplus package of R^30^ using Conover’s post hoc test^31^ for the pairwise comparisons. Comparisons between PD‐M and HC groups in the validation cohort were made using T test.

#### Logistic regression

Differentiations between groups were analyzed by forced entry logistic regression of variables, using SPSS software. α‐Syn forms with *P* values ≤ 0.05 were selected and a constant parameter was calculated to each variable. According to the equation, a personalized score (Z) was calculated and used to yield a predicted probability of PD. A receiver operating characteristic (ROC) curve was calculated according to the predicted probabilities. The distribution of values of the two variables, total and PK^res^ α‐Syn, was found to be not significantly different from a normal distribution of values (*P* = 0.121 and p = 0.261, respectively. Kolmogorov–Smirnov test). Whereas the PSer129 α‐Syn was borderline, with a *P*‐value = 0.048. A log transformation in the case of PSer129 α‐Syn gave a *P*‐value = 0.511. Hosmer–Lemeshow test gave a χ^2^ of 8.033; with 8 degrees of freedom, that corresponds to a *P*‐value of 0.430. Indicating that the model is well calibrated.

All data were analyzed using a qualified statistical software package (SPSS for Windows, Version 25.0, SPSS Inc., Chicago, Illinois, USA). A *P*‐value of less than 0.05 was considered significant. Prism8 (GraphPad) was used for graphics and calculations of Pearson’s correlations.

## Results

### Demographic features

Demographic and clinical data for the BioFIND (discovery) cohort comprising 45 HC and 46 PD participants are listed in Table 1. Age, race, and education similarly varied between the groups. The BioFIND PD group was subdivided according to presentation of symptoms to PD‐motor (PD‐M) and PD with cognitive impairment (PD‐D), represented by MoCA ≤ 25.

**Table 1:** Demographic and clinical features in BioFIND (discovery) cohort.

Variable	PD‐Motor (*n* = 32)	PD‐Dementia (*n* = 14)	Healthy Control (*n* = 45)
Age			
Mean (SD)	70 (6.0)	74.1 (7.6)	69.5 (8.3)
(Min, Max)	(60,83)	(59, 84)	(57,96)
Gender			
Male	20 (62%)	11(78%)	19 (42%)
Female	12 (38%)	3 (22%)	26 (58%)
Ethnicity			
Hispanic/Latino	1(3%)	0 (0%)	3 (6%)
Not Hispanic/Latino	31 (97%)	14 (100%)	42 (94%)
Race			
White	32 (100%)	14 (100%)	38 (87%)
African‐American	0 (0%)	0 (0%)	5 (11%)
other	0 (0%)	0 (0%)	2(2%)
Education			
<13 years	2 (6%)	2 (14%)	3 (7%)
>13 years	30 (94%)	12 (86%)	42 (93%)
Family history			
Positive	13 (40%)	2 (14%)	5 (11%)
Negative	19 (60%)	12 (86%)	40 (89%)
Disease duration			
Mean (SD)	6 (3.4)	7 (3.6)	N/A
(Min, Max)	(1, 15)	(3,15)	N/A
PD Medication			
On PD medication	32 (100%)	12 (85%)	N/A
L‐Dopa	26 (81%)	12 (85%)	N/A
Dopamine Agonist	18 (56%)	6 (42%)	N/A
MDS‐UPDRS			
MDS‐UDPRS total	53.5(19.5)	59.3 (31.7)	N/A
MDS‐UDPRS I	3.0 (2.5)	4.3 (2.5)	N/A
MDS‐UDPRS II	10.5 (5.7)	12.0 (5.7)	N/A
MDS‐UDPRS III	37.6 (14)	38.3 (14)	N/A
MDS‐UDPRS IV	2.5 (2.6)	4.6 (2.6)	N/A
H&Y			
Stage 1‐2	18 (56%)	11 (79%)	N/A
Stage 3	10 (31%)	2 (14%)	N/A
Stage 4	4 (13%)	1 (7%)	N/A
Stage 5	0 (0%)	0 (0%)	N/A
MoCA			
Mean (SD)	28.1 (1.3)	23.0 (2.1)	27.1 (1.4)
(Min, Max)	(26, 30)	(18, 25)	(26, 30)
REM			
Negative (< 5)	20 (63%)	7 (50%)	38 (84%)
Positive (≥5)	12 (37%)	7 (50%)	7 (16%)

Family history: at least one affected family member (positive); or no affected family member (negative); MDS‐UPDRS, Movement Disorders Society—Unified Parkinson’s Disease Rating Scale Motor score, mean (SD); H&Y, Hoehn and Yahr; MoCA, Montreal Cognitive Assessment; N/A, not applicable; REM Sleep Behavior Disorder Screening Questionnaire.

### Higher levels of total α‐Syn in PD‐M than HC group

**Figure 1:**
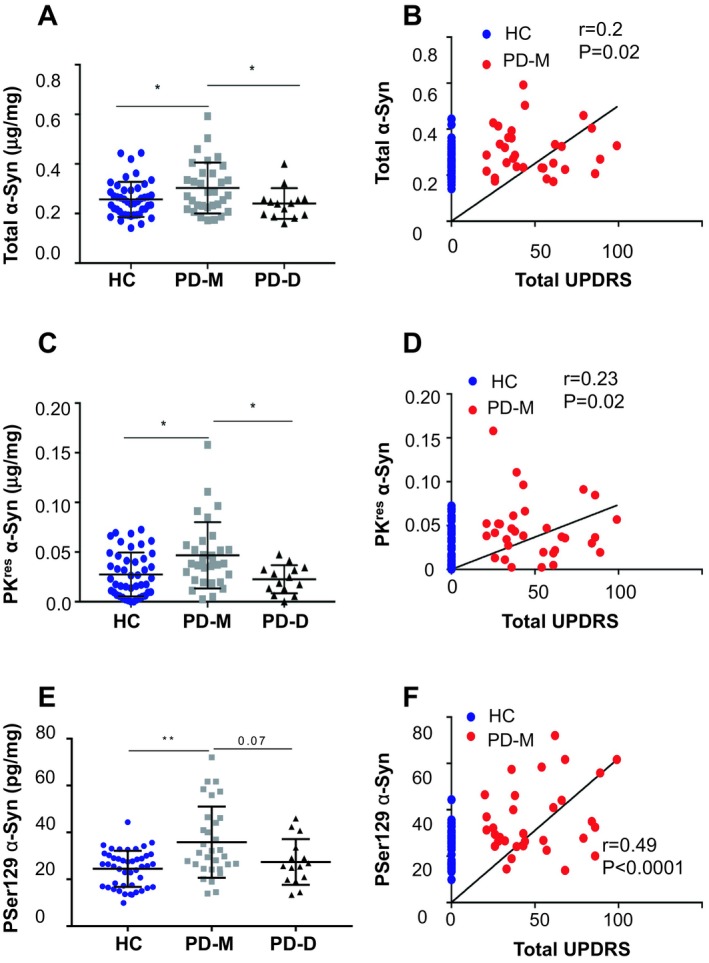
α‐Syn levels in samples of whole blood cells determined by Lipid ELISA. Graph showing mean ± SD of total α‐Syn (A), PK^res^ α‐Syn (C) or PSer129 α‐Syn (E) detected through binding to a mixture of PI:PS:PE:GM1 (1:1:1:1) that were immobilized to the ELISA plate using methanol or cyclohexene as a solvent. Pearson’s correlation coefficient for total α‐Syn (B), PK^res^ α‐Syn (D) and PSer129 α‐Syn (F) with total UPDRS (I + II+III) scores. HC, healthy controls; PD‐M, PD with motor symptoms; PD‐D, PD with cognitive symptoms. *P < 0.05; **P < 0.01 (Kruskal–Wallis test).

Total α‐Syn levels were determined in BioFIND samples of lysed blood cell pellets, in two independent analyses. The average amount of total α‐Syn (analyses 1 and 2, see methods) determined in HC samples, 0.261 ± 0.06 μg α‐Syn/mg protein, was somewhat lower (*P* = 0.07, Kruskal–Wallis) than the amounts determined in the entire PD group (0.286 ± 0.09 μg α‐Syn/mg protein). However, excluding PD‐D samples, defined by MoCA ≤ 25, resulted in a significant difference between a PD‐M (0.307 ± 0.10 μg α‐Syn/mg protein) and HC group (*P* = 0.02, Kruskal–Wallis; Fig. 1A). Pearson’s correlation coefficient between total α‐Syn levels in the PD‐M group and disease severity, represented by UPDRS, resulted in *r* = 0.2 and *P* = 0.02 (Fig. 1B). Interestingly, total α‐Syn levels differed between the two PD subgroups. The levels determined in the PD‐D group (0.241 ± 0.05 μg α‐Syn/mg protein) were significantly lower than the PD‐M group (*P* = 0.01 Kruskal–Wallis; Fig. 1A and Table 2). Importantly, closely similar levels of total α‐Syn were detected in both measurements (Table 2; *r* = 0.5; *P* value of correlation < 0.0001), showing repeatability of the method.

**Table 2:** Concentrations determined in BioFIND (discovery) cohort.

α‐Syn form^a^	HC (*n* = 45)	PD‐M (*n* = 32)	PD‐D (*n* = 14)	P (PD‐M vs HC)^i^	P (PD‐M vs PD‐D)^i^
Total (methanol) α‐Syn^b^					
Average	0.261 ± 0.06	0.307 ± 0.10	0.241 ± 0.05	0.02	0.01
Analysis 1	0.249 ± 0.09	0.304 ± 0.12	0.239 ± 0.05	0.04	0.06
Analysis 2	0.273 ± 0.07	0.317 ± 0.09	0.241 ± 0.06	0.06	0.01
Total (cyclohexene) α‐Syn^b^	0.55 ± 0.12	0.56 ± 0.11	0.58 ± 0.13	ns	ns
PK^res^ α‐Syn^b^					
Average	0.030 ± 0.02	0.044 ± 0.03	0.024 ± 0.01	0.01	0.01
Analysis 1	0.032 ± 0.03	0.048 ± 0.03	0.028 ± 0.02	0.01	0.04
Analysis 2	0.028 ± 0.02	0.040 ± 0.03	0.020 ± 0.01	0.04	0.03

α‐Syn form^c^	HC (n = 45)	PD‐M (n = 32)	PD‐D (n = 14)	P (PD‐M vs HC)	P (PD‐M vs PD‐D)
PSer129^d^	24.480 ± 7.60	35.820 ± 15.19	27.370 ± 9.76	0.001	0.07
Oxidized α‐Syn^b^	0.022 ± 0.01	0.024 ± 0.09	0.021 ± 0.07	ns	ns

Blood parameters	HC (n = 45)	PD‐M (n = 32)	PD‐D (n = 14)	P (PD‐M vs HC)	P (PD‐M vs PD‐D)
Protein^e^	87.890 ± 17.50	80.080 ± 15.30	93.810 ± 14.30	ns	ns
Hemoglobin^f^	0.026 ± 0.01	0.027 ± 0.01	0.022 ± 0.00	ns	ns
Iron^g^	0.046 ± 0.01	0.044 ± 0.01	0.047 ± 0.01	ns	ns
H‐ferritin^h^	2.870 ± 1.40	2.490 ± 1.30	3.580 ± 3.59	ns	ns

a mean ± SD

b µg/mg protein

c hemoglobin clearance with HemoVoid

d pg/mg protein

e mg/ml lysed blood cell pellets

f mg/mg protein

g μg/mg protein

h ng/mg protein

i Kruskal–Wallis. Protein levels determined per volume of lysed blood cell pellet samples. The concentrations of all other variables presented per mg total proteins (mean ± SD). *P* value calculated by Kruskal–Wallis and Conover’s post hoc test for the pairwise comparisons; ns, not significant.

Total α‐Syn levels were determined one more time (analysis 3) however with a slight modification in the protocol. Cyclohexene was used to solubilize the lipids before their immobilization on the ELISA plate. The rationale for testing cyclohexene (instead of methanol) is that different solvents may induce the formation of different lipid structures during solvent evaporation and therefore, affect α‐Syn binding and detection by the Lipid‐ELISA assay. Total α‐Syn determined in HC samples was 0.55 ± 0.12 μg α‐Syn/mg protein, which is ~twofold higher than α‐Syn levels determined with methanol as a solvent for the lipids. Similar results, showing ~twofold higher total α‐Syn levels were detected also in the PD groups. However, no significant differences were detected between the HC and PD groups using this solvent (Table 2).

### Higher levels of proteinase K‐resistant (PK^res^) α‐Syn in PD‐M than HC group

PK^res^ α‐Syn levels^17^ were determined in BioFind samples in two independent analyses (analyses 1 and 2, see methods). The average PK^res^ α‐Syn levels in the HC group, determined in the two analyses (0.030 ± 0.02 μg α‐Syn/mg protein) is significantly lower than the amounts determined in the PD‐M group (0.044 ± 0.03 μg α‐Syn/mg protein; *P* = 0.01, Kruskal–Wallis). In addition, PK^res^ α‐Syn levels significantly differ between PD‐M and PD‐D (0.024 ± 0.01 μg α‐Syn/mg protein) groups (*P* = 0.01, Kruskal–Wallis; Fig. 1C and Table 2). Pearson’s correlation coefficient between PK^res^ α‐Syn levels in the PD‐M group and disease severity, represented by UPDRS, resulted in *r* = 0.23 and *P* = 0.02 (Fig. 1D). Importantly, closely similar PK^res^ α‐Syn values were determined in both analyses (Table 2; *r* = 0.6; *P* value of correlation < 0.0001).

### Higher levels of phospho Serine (PSer) 129 α‐Syn in PD‐M than HC group

Aiming at improving the classification rate between the groups, we determined the levels of PSer129 α‐Syn, a pathogenic form of α‐Syn. PSer129 α‐Syn was shown to bind membrane lipids^32^ and therefore can be determined by the Lipid‐ELISA assay. PSer129 α‐Syn levels in blood cells are considerably lower than total α‐Syn and occur at the picogram (pg) scale. To enable its detection, samples were treated to remove hemoglobin using HemoVoid and detection was performed using cyclohexene for lipid immobilization.

PSer129 α‐Syn levels determined in the HC group were 24.48 ± 7.6 pg α‐Syn/mg protein. These levels are significantly lower than the levels detected in the PD‐M group, 35.82 ± 15.19 pg α‐Syn/mg protein (*P* value = 0.001; Kruskal–Wallis; Fig. 1E and Table 2). PSer129 α‐Syn levels in the PD‐M group correlate with disease severity, represented by UPDRS, with *r* = 0.49 and *P* < 0.0001 (Fig. 1F). Interestingly, PSer129 α‐Syn levels determined in the PD‐D group, 27.37 ± 9.76 pg α‐Syn/mg protein (*n* = 14) showed a tendency for a difference with PD‐M group (*P* = 0.07, Kruskal–Wallis).

### Oxidized α‐Syn in blood cells

Oxidized α‐Syn levels, detected with the syn303 antibody,^33^ were determined in samples pre‐treated with HemoVoid. Samples were applied on microtitter plates, pre‐coated with lipids dissolved in cyclohexene. We detected 0.022 ± 0.012 μg oxidized α‐Syn/mg protein in the HC group. These levels did not differ from the levels detected in the PD‐M or PD‐D groups (Table 2).

### General blood variables

The concentrations of total protein, iron, hemoglobin and H‐ferritin were determined as indicators of quality of the BioFIND samples and as potential variables to support differentiation between the groups. The detected concentrations varied within groups, with no significant differences between the groups (Table 2).

### α‐Syn forms differentiate HC and PD groups

Based on the concentrations of total α‐Syn (average of analyses 1 and 2), PK^res^ α‐Syn (average of analyses 1 and 2) and PSer129 α‐Syn, determined in BioFIND samples, we performed forced entry logistic regression analysis (Table 3). These variables were used to develop a composite biomarker for differentiating PD‐M and healthy controls. A specific Z‐value that is calculated using the following equation:

$$Z=-5.007+\left(0.095\times PSer129\alpha -\mathrm{Syn}\right)+\left(3.203\times total\alpha -Syn\right)+\left(28.107\times {\mathrm{PK}}^{\mathrm{res}}\alpha -Syn\right)$$

**Table 3:** Logistic regression model summary.

Variable	B	S.E.	Sig.
Total α‐Syn	3.203	3.266	0.327
PKres α‐Syn	28.107	11.881	0.018
PSer129 α‐Syn	0.095	0.028	0.001
Constant	−5.007	1.284	>0.00001

The equation is used to calculate P (predict), a value used to determine the degree of classification between the test groups.

$$P\left(\text{predict}\right)=\frac{1}{1+e^{-Z}}$$

Cut off was set at = 0.5, where *P* (predict) < 0.5 is HC and *P* (predict)> 0.5 is PD.

**Figure 2:**
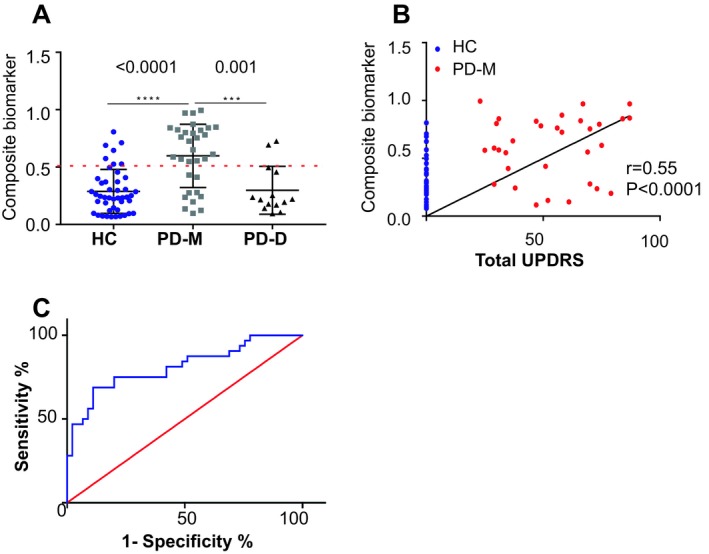
A composite biomarker differentiates PD with motor symptoms (PD‐M) and healthy controls (HC) in the BioFIND (discovery) cohort. (A) Graph showing the classification of the composite biomarker, consisting of the concentrations of total α‐Syn, PK^res^ and PSer129 α‐Syn, combined with logistic regression as determined in HC (*n* = 45), PD‐M (*n* = 32), and PD‐D (*n* = 14) groups. (B) The composite biomarker in PD‐M correlates with UPDRS (I + II+III). (C) ROC curve showing the strength of the composite biomarker in differentiating the PD‐M and HC groups.

The composite biomarker demonstrates a meaningful degree of disease classification (Fig. 2A); correlates with disease severity (UPDRS), with *r* = 0.55 and *P* < 0.0001 (Fig. 2B); and provides an AUC = 0.81 with 75% sensitivity and 80% specificity (Fig. 2C).

In addition, the composite biomarker demonstrates an AUC = 0.80 between BioFind PD subgroups, PD‐M and PD‐D. This degree of classification between the PD subgroups does not allow classification, yet, it provides an indication for a biochemical difference between these groups.

### Validation assay for the composite biomarker

**Figure 3:**
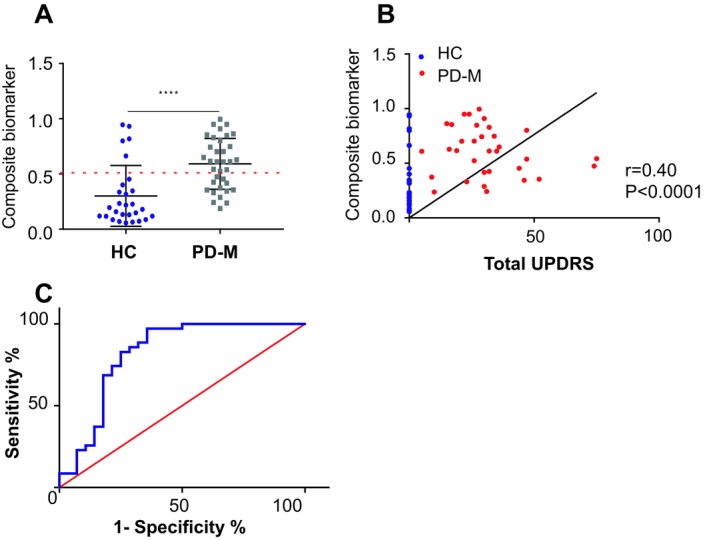
A composite biomarker differentiates PD‐M and healthy controls (HC) in the validation cohort. (A) Graph showing the classification of the composite biomarker, consisting of the concentrations of total α‐Syn, PK^res^ and PSer129 α‐Syn, combined with logistic regression as determined in HC (*n* = 28) and PD‐M (*n* = 35). (B) Pearson’s correlation coefficient for the composite biomarker in PD‐M with UPDRS (I + II+III). (C) ROC curve showing the strength of the composite biomarker in differentiating the PD‐M and HC groups.

The performance of the composite biomarker was next validated using samples collected at the neurology department at Hadassah hospital, comprising 28 HC and 35 PD‐M participants (Table 4). Levels of total α‐Syn, PK^res^ α‐Syn and PSer129 were determined (Table 5). Placing the concentrations of these α‐Syn variables in the equation (as above) resulted in an AUC = 0.81 with 63% sensitivity and 82% specificity (Fig. 3). Finally, the Pearson’s correlation coefficient of the composite biomarker and disease severity (UPDRS) provided *r* = 0.4 and *P* < 0.001 (Fig. 3).

**Table 4:** Demographic and clinical features in Hadassah (validation) cohort.

Variable	PD‐Motor (*n* = 35)	Healthy Control (*n* = 28)
Age		
Mean (SD)	70.9 (7.3)	68.3 (8.5)
(Min, Max)	(53, 83)	(54, 84)
Gender		
Male	17 (48.5%)	15 (53.5%)
Female	18 (51.5 %)	13 (46.5%)
Disease duration		
Mean (SD)	5.7 (4.5)	N/A
(Min, Max)	(1,17)	N/A
PD Medication		N/A
On PD medication	35 (100%)	N/A
L‐Dopa	23 (65%)	N/A
Dopamine Agonist	5 (14%)	N/A
MDS‐UPDRS		
MDS‐UDPRS total	31.80 (19.0)	N/A
MDS‐UDPRS I	1.03 (1.2)	N/A
MDS‐UDPRS II	9.06 (5.9)	N/A
MDS‐UDPRS III	20.03 (12.0)	N/A
MDS‐UDPRS IV	2.64 (3.4)	N/A
H&Y		
Stage 1–2	27 (78%)	N/A
Stage 3	8 (22%)	N/A
Stage 4	0 (0%)	N/A
Stage 5	0 (0%)	N/A
MoCA		
MoCA> 25	35 (100%)	28 (100%)
MoCA < 25	0 (0%)	0 (0%)

MDS‐UPDRS, Movement Disorders Society—Unified Parkinson’s Disease Rating Scale Motor score, mean (SD); H&Y, Hoehn and Yahr; MoCA, Montreal Cognitive Assessment; N/A, not applicable.

**Table 5:** Concentrations determined in Hadassah (validation) cohort.

	HC (*n* = 28)	PD‐M (*n* = 35)	P (PD‐M vs HC)
α‐ Syn form^a^			
Total (methanol) α‐Syn^b^	0.253 ± 0.05	0.340 ± 0.12	**0.001
PK^res^ α‐Syn^b^	0.031 ± 0.03	0.055 ± 0.03	**0.001
PSer129^c^,^d^	24.110 ± 8.90	30.930 ± 8.50	**0.003
Blood parameters			
Protein^e^	77.330 ± 14.90	72.190 ± 10.40	ns
Hemoglobin^f^	0.108 ± 0.02	0.105 ± 0.02	ns
H‐ferritin^g^	2.720 ± 0.81	2.600 ± 1.01	ns

a mean ± SD

b µg/mg protein

c hemoglobin clearance with HemoVoid

d pg/mg protein

e mg/ml lysed blood cell pellets

f mg/mg protein.

g ng/mg protein.

## Discussion

In this cross‐section study we test the usefulness of blood cells expressed α‐Syn as a biomarker for PD. The concentrations of total α‐Syn and two additional α‐Syn forms, PK^res^ and PSer129 were determined by a lipid‐ELISA assay and found to significantly differ between healthy controls and individuals affected with PD, presenting motor symptoms without dementia (PD‐M). These three α‐Syn variables were used to develop a predictive model capable of differentiating PD‐M and control groups, with a calculated AUC (95% CI) of 0.81 (0.71 to 0.91) and a considerable specificity value (80%). Validation of the predictive model in a second cohort resulted in highly similar classification rate between HC and the PD‐M groups. In addition, our results suggest differences in performance of the composite biomarker between PD subgroups, PD‐M and PD‐D. We conclude that blood cells expressed α‐Syn can differentiate PD‐M and HC with a high degree of accuracy. It provides a reliable classification rate, correlates with the severity of disease and is reproducible. However, additional developments in the identified composite biomarker are needed to improve its performance.

We determined α‐Syn levels using a Lipid ELISA assays^17^ in which α‐Syn capture is enabled using immobilized lipids. α‐Syn capture by lipids may represent either an advantage or a disadvantage over capture with specific anti α‐Syn antibodies. For example, lipid binding may enhance structure acquisition of the soluble α‐Syn protein and improve its detection by antibodies. However, α‐Syn capture by the Lipid ELISA is limited to the detection of lipid binding α‐Syn forms. Moreover, because detergents may interfere with the lipids in the assay the detection is limited to soluble α‐Syn forms. Of relevance, previous studies reported differential levels of phosphorylated, nitrated, glycated, SUMOlated, oligomeric/aggregated and membrane‐bound α‐Syn forms in blood cells of PD and HC donors.^14^, ^18^ The occurrence of toxic α‐Syn forms, including oligomeric α‐Syn, in the blood and the reports indicating their lipid‐binding properties^34^ provides opportunities for further developments that will potentially improve the accuracy of PD diagnosis.

A useful biomarker will assist diagnosis of PD. On the other hand, the development of a useful biomarker will benefit better classification of the disease during recruitment of study cohorts. PD may potentially consist of subtypes, including variable symptoms, response to therapy, rate of disease progression and genetics.^2^ A specific biomarker may present compatibility with a specific disease subtype. It is plausible that testing the diagnostic efficacy of the identified composite biomarker in blood samples of patients that their Dopamine Transporter Single Photon Emission Computed Tomography (DAT‐SPECT) in putamen shows affected dopaminergic neuronal cells,^35^ will improve the accuracy of our model.

Tracking longitudinally the dynamics of the composite biomarker with progression of Parkinson’s disease is critical for its evaluation as a reliable biomarker that reflects on severity of disease. Previous studies have shown that α‐Syn levels, determined in CSF or blood plasma, differentiated between early PD and control groups.^16^, ^35^, ^36^, ^37^, ^38^ However, a recent longitudinal study found no correlation between CSF α‐Syn levels and progression in motor or non‐motor symptoms of PD.s^38^ Therefore, a longitudinal study that will determine whether alterations in blood cell‐expressed α‐Syn forms are associated with disease progression is required.

## Conflict of Interest

Authors S.A.E., J.G., M.P., P.A. and D.A have no conflict of interests. R.S. owns patent PCT/IL2014/050191: An ELISA method for a sensitive detection of alpha synuclein, consisting of its phospholipids binding properties.

## Authors' contributions

Suaad Abd Elhadi and Jessica Grigoletto: analyzed samples and data; Maura Poli and Paolo Arosio developed protocols; David Arkadir examined patients; Ronit Sharon developed study concept and wrote the manuscript. All authors read and approved the final manuscript.

## Supporting information


**Figure S1.** Sensitivity and specificity of PSer129 α‐Syn and oxidized α‐Syn detection

**Appendix S1.** Assay sensitivity and specificity
