# Supplementary figures and images for: α‐Synuclein in blood cells differentiates Parkinson’s disease from healthy controls
Source: Ann Clin Transl Neurol. 2019 Nov 19;6(12):2426–36. doi: 10.1002/acn3.50944 (PMC6917335; doi:10.1002/acn3.50944)

Fig. S1

**A**

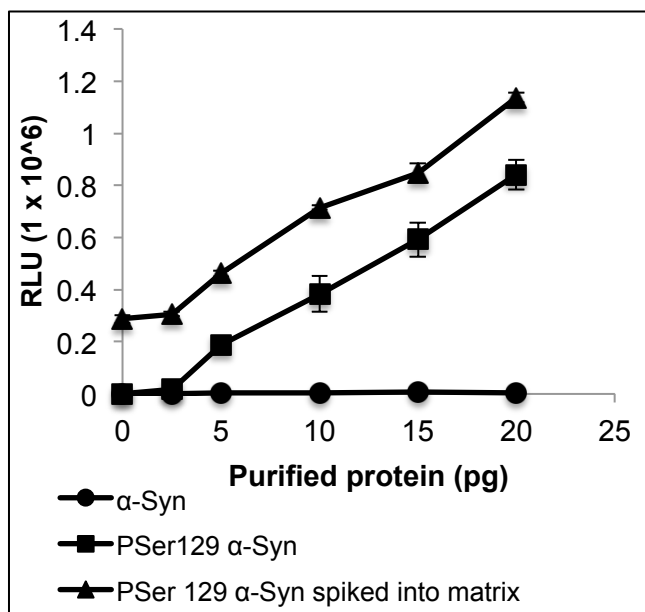

**B**

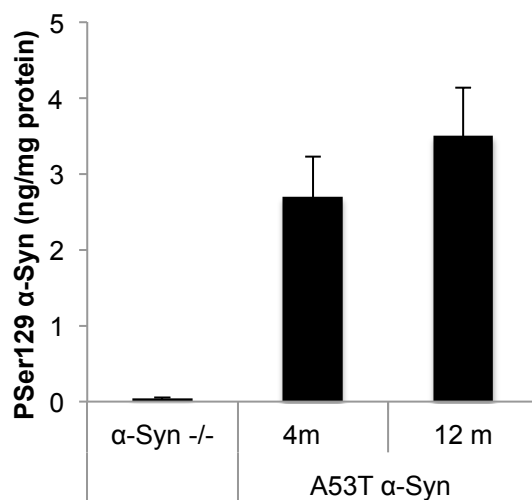

**C**

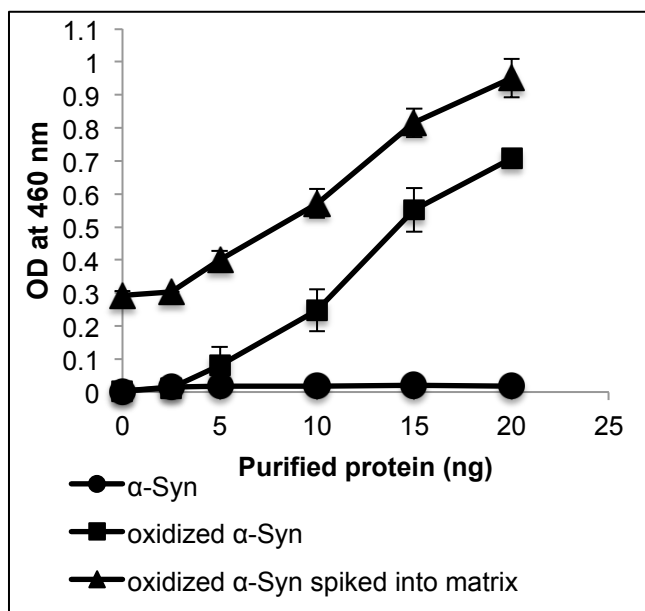

**D**

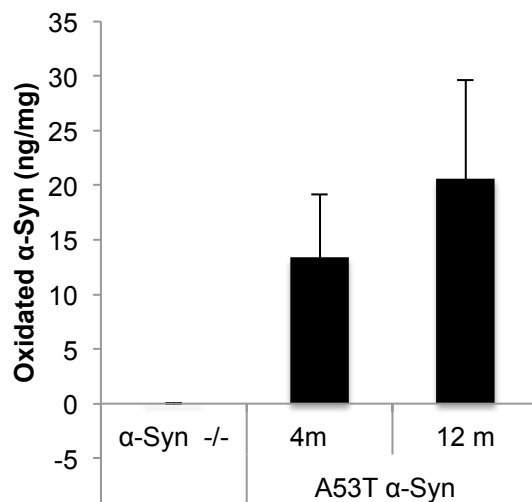

Supplement: Supplementary file 1 — Figure S1. Sensitivity and specificity of PSer129 α‐Syn and oxidized α‐Syn detection [file ACN3-6-2426-s001.pdf]
