# Supplementary material for: α‐Synuclein in blood cells differentiates Parkinson’s disease from healthy controls
Source: Ann Clin Transl Neurol. 2019 Nov 19;6(12):2426–36. doi: 10.1002/acn3.50944 (PMC6917335; doi:10.1002/acn3.50944)
Supplement: Supplementary file 2 — Appendix S1. Assay sensitivity and specificity [file ACN3-6-2426-s002.docx]

Assay sensitivity and specificity

The specific detection of PSer129 α-Syn is demonstrated using purified recombinant α-Syn proteins, e.g., human wt α-Syn and PSer129 (Proteos, Kalamazoo, US). The proteins were applied at 0-20 pg per well. The results (Appendix 1A) show a linear, dose-dependent detection of PSer129 α-Syn using the anti PSer129 ab, pSyn#64 (WAKO). Importantly, no specific signal could be detected for the purified, non phosphorylated α-Syn protein with this antibody. Matrix effect on PSer129 α-Syn detection was tested by spiking the purified PSer129 α-Syn protein (at 0-20 pg/well) into a sample of blood, pre-treated with HemoVoid (the equivalent amount of 0.16 mg protein before HemoVoid pre-treatment; see method). The results show closely similar detection levels of PSer129 α-Syn in the presence and absence of matrix. With 96% recovery (calculated as PSer129 α-Syn spiked in sample – sample alone) /(spiked PSer129 α-Syn)*100%). The minimal detectable PSer 129 α-Syn level was determined as the amount of PSer129 α-Syn that corresponded to a value that was at least two standard deviations above the background, set at 5 pg PSer129α-Syn. Detection of PSer129 α-Syn was verified in A53T α-Syn tg mouse brains ^33^. Samples of whole mouse brain extracts ^40^ from young (4 months) and old (12 months) mice (n=4) were applied at 0-4 µg protein, in the Lipid-ELISA plate, in triplicates. Background levels were determined using samples of whole brain extracts from α-Syn -/- mouse brains ^41^ (n=4). Higher levels of Pser129 α-Syn were detected in the old (~3.5 ng PSer 129 α-Syn/mg protein sample) than the young (~2.7 ng PSer129 α-Syn/mg protein) mouse brain extract (Appendix 1B).

The specific detection of oxidized α-Syn is demonstrated using a standard curve consisting of a recombinant α-Syn protein that was pre-treated with ferrous sulfate (0.5 mM) and H_2_O_2_ (1mM) ^29^ or left untreated. The purified proteins were applied at 0-20 ng per well, in triplicates. The results (Appendix 1C) indicate a linear detection curve for oxidized α-Syn using the syn303 monoclonal antibody. The minimal detectable oxidized α-Syn level was set at 5 ng as above. Matrix interference was tested by spiking the oxidized α-Syn protein into a blood sample, that was pre-treated with HemoVoid (see method). No effect for matrix on oxidized α-Syn could be detected as indicated by the calculated 93% recovery rate. The levels of oxidized α-Syn were next determined in whole brain extracts of A53T α-Syn tg mice. Oxidized α-Syn levels, detected in young (4 months) and old (12 months) mice, were ~13.3 and ~20.3 ng α-Syn/mg protein sample, respectively (Appendix 1D). Background levels were determined using whole brain extracts of α-Syn-/- mice (n=4).
